# Renal function mediates the association between neutrophil percentage-to-albumin ratio and survival in cancer survivors: a large cross-sectional study

**DOI:** 10.3389/fonc.2025.1620484

**Published:** 2026-01-14

**Authors:** Weiming Chen, Wenjing Cai, Hao Zeng, Zhisheng Wang, Xin Luo, Zengkai Xu, Jiahuang Wu, Yong Zhu, Hongjin Wang

**Affiliations:** 1 Department of Cardio-Thoracic Surgery, Longyan First Affiliated Hospital of Fujian Medical University, Longyan, China; 2 Department of Thoracic Surgery, Fujian Medical University Union Hospital, Fuzhou, China; 3 Fujian Medical University, Fuzhou, China; 4 Department of Gastroenterology and Anorectal Surgery, Longyan First Affiliated Hospital of Fujian Medical University, Longyan, China

**Keywords:** neutrophil percentage to albumin ratio, cancer survivors, cancer-specific mortality, all-cause mortality, renal function

## Abstract

**Background:**

Cancer survivors face heightened mortality risks due to recurrence, comorbidities, and insufficient prognostic tools. The neutrophil percentage-to-albumin ratio (NPAR), integrating inflammation and nutrition, has shown prognostic value in cancers but lacks validation in survivor populations.

**Objective:**

To assess the predictive efficacy of NPAR on mortality in cancer survivors and to explore the mediating role of renal function.

**Methods:**

Data from NHANES (2003-2018) were used to analyse 3,134 cancer survivors. Cox models assessed the association of NPAR with all-cause, cancer-specific, and non-cancer mortality. Receiver operating characteristic (ROC) curve analysis was used to evaluate the predictive performance of NPAR. Restricted cubic splines assessed non-linear associations, while mediation analysis quantified the role of renal function. The external validation study was conducted between January 2016 and December 2018, involving an additional 985 cancer patients recruited from a tertiary hospital in China.

**Results:**

This study demonstrated that each 1-unit increase in NPAR was associated with a 10% increase in the risk of all-cause mortality, a 6% increase in the risk of cancer-related mortality, and a 12% increase in the risk of non-cancer mortality. This dose-dependent association remained robust in multivariable-adjusted models. RCS analyses further revealed a nonlinear relationship between NPAR and risk of death, with a steep inflection point in risk of all-cause mortality when NPAR exceeded 12.84. ROC analysis showed that NPAR had an AUC of 0.608 for all-cause mortality, outperforming the systemic immune-inflammation index (SII). Finally, mediation analyses elucidated renal impairment as a key pathway through which NPAR affects prognosis: 23.08% of the NPAR-mediated risk of all-cause mortality was driven by a decline in eGFR, and 21.97% of the risk of non-cancer mortality was attributable to worsening renal function. In real-world data analysis, NPAR has also been demonstrated to correlate positively with all-cause mortality, cancer-specific mortality, and non-cancer-specific mortality among cancer patients.

**Conclusion:**

NPAR is a robust prognostic biomarker for mortality in cancer survivors, mediated in part by renal dysfunction. These findings highlight the clinical utility of NPAR and the need for interventions targeting the inflammation-nutrition-kidney pathways.

## Introduction

Cancer poses a significant global public health threat, with its disease burden on the rise. In 2022, there are expected to be around 20 million new cancer cases and 10 million deaths worldwide. Projections indicate that by 2050, the annual number of new cases will increase by 77%, reaching 35 million (1). Although targeted therapy and immunotherapy have revolutionized cancer treatment, increasing the overall five-year relative survival rate to 68% (2), survivors still face many complex challenges, including the risk of recurrence, uncertainty about death, and secondary complications caused by psychological barriers (3). In this context, constructing a precise prognostic prediction system has become the focus of clinical research. In previous studies, the mechanism by which inflammation is associated with cancer progression and risk of death has been widely confirmed (4, 5). The neutrophil-to-lymphocyte ratio (NLR), the platelet-to-lymphocyte ratio(PLR) (6), the systemic immune-inflammation index(SII) (7), the albumin-to-globulin ratio (AGR), and the prognostic nutritional index (PNI) (8) have all been shown to correlate with tumor prognosis.

It is worth noting that nutritional status is involved in the cancer process through bidirectional regulation of pro-inflammatory/anti-inflammatory, suggesting a complex interaction between inflammation-nutrition-cancer (9). Based on this, researchers are systematically integrating inflammatory and nutritional indicators to construct a new assessment system: neutrophils, as a core component of innate immunity, play a dual role in the tumor microenvironment, both anti-tumor and pro-cancer (10, 11); albumin, as a classic nutritional marker, its dynamic changes have important predictive value for disease prognosis (12). The Bernard team innovatively proposed the neutrophil-albumin ratio (NPAR) concept, which has been shown to be significantly effective in predicting the response to neoadjuvant therapy in colorectal cancer (13). Subsequent studies have further verified the universality of NPAR for prognostic assessment of oral cancer (14), gastrointestinal tumors (15), breast cancer (16), lung cancer (17), pancreatic cancer (18), and bladder cancer. Among cancer patients, the occurrence of cachexia is common; this syndrome of malnutrition is associated with cancer, chronic kidney failure, and other chronic diseases (19). Additionally, the incidence of acute and chronic kidney failure in cancer patients is often associated with mortality (20). However, there is currently a lack of research on the correlation between nutrition, kidney function, and cancer.

To address this critical knowledge gap, this study systematically examined the predictive efficacy of NPAR for all-cause mortality and cause-specific mortality in cancer survivors, utilizing long-term follow-up data from the 2003–2018 cohort of the National Health and Nutrition Examination Survey (NHANES). Notably, this study innovatively introduces the analysis of mediation effects, revealing for the first time the mediating mechanism of renal function indicators between NPAR and mortality, thereby providing a multi-dimensional theoretical foundation for the clinical application of NPAR as a new prognostic biomarker.

## Materials and methods

### Data source

This study used a retrospective cohort design, with data from the NHANES hosted by the US Centers for Disease Control and Prevention (CDC). This database collects the health status, lifestyle and laboratory test indicators of non-hospitalised adult populations in the United States through stratified multi-stage probability sampling methods, and is nationally representative. All procedures involving human participants in the original NHANES surveys were conducted in accordance with the ethical standards of the National Center for Health Statistics (NCHS) Research Ethics Review Board (ERB) and with the 1964 Helsinki Declaration and its later amendments or comparable ethical standards.

Written informed consent was obtained from all NHANES participants prior to their inclusion in the survey. Detailed information regarding the NHANES informed consent process is publicly available on the NHANES website.

The protocol for each NHANES cycle is reviewed and approved annually by the NCHS Research Ethics Review Board. Because this present study involved the secondary analysis of existing, anonymized data, it was considered exempt from requiring additional ERB. All analyses were performed in accordance with relevant guidelines and regulations.

### Study population

**Figure 1:**
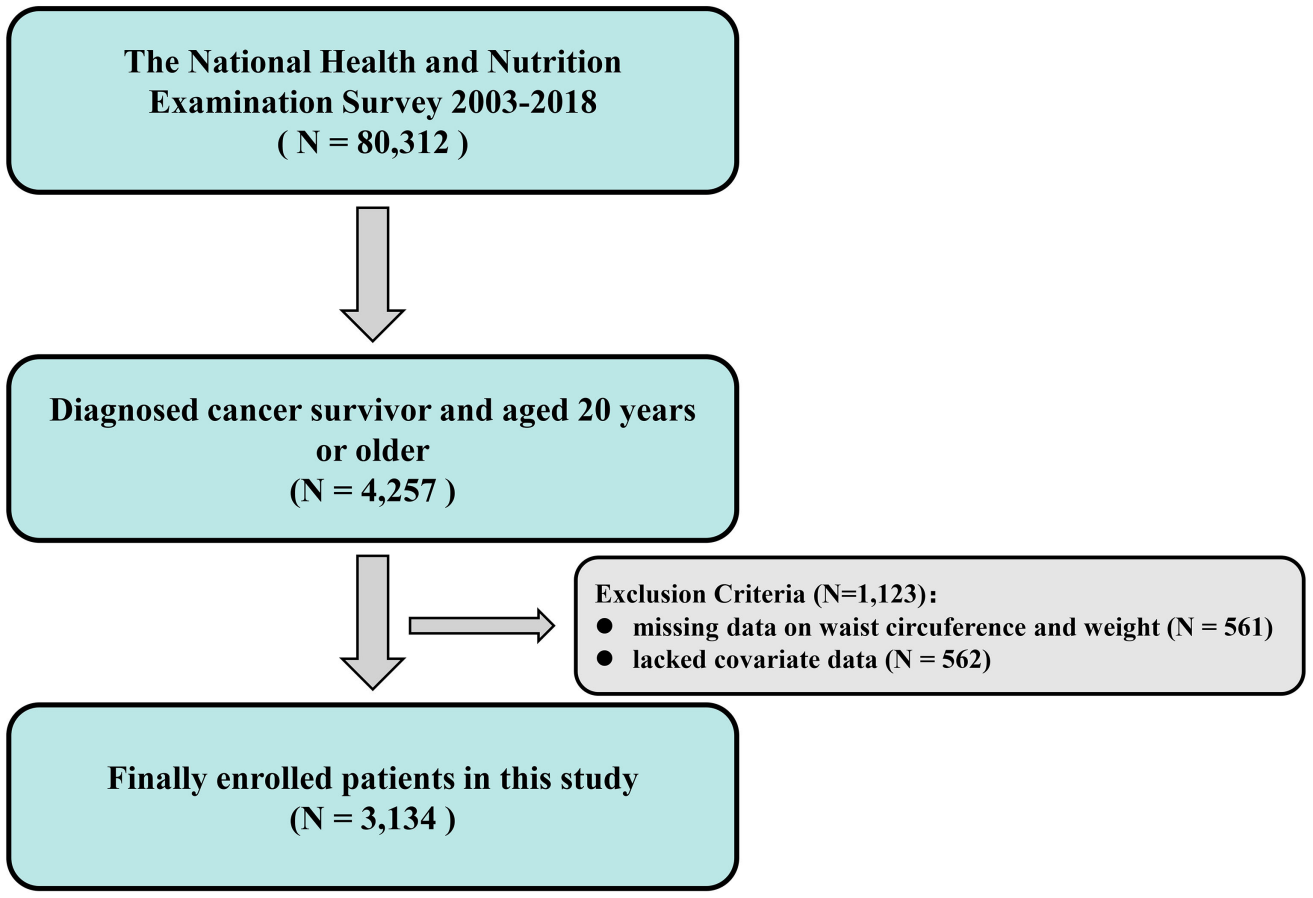
Flowchart of sample selection process.

This study strictly screened cancer survivors who met the following inclusion criteria: (1) age ≥ 20 years; (2) diagnosed with cancer survivor status by NHANES; (3) complete NPAR calculation parameters (neutrophil percentage and serum albumin test values) and serum creatinine. Exclusion criteria included: (1) missing key covariates (demographic characteristics or comorbidity data); and (2) incomplete follow-up information or insufficient follow-up periods. After rigorous screening, the final cohort included 3,134 cancer survivors who met the study requirements (see **Figure 1** for the sample selection process).

### NPAR measurement

The NPAR is calculated as the quotient of the neutrophil percentage and the serum albumin concentration (g/dl). This indicator simultaneously reflects the body’s inflammatory activation and nutritional reserve status. Subjects were divided into four quartile groups (Q1 ≤ 25%<Q2 ≤ 50%<Q3 ≤ 75%<Q4) according to baseline NPAR.

### Outcomes

Cancer survivors were identified through the NHANES questionnaire, which asked, “Has a doctor or other health professional ever told you that you have cancer?” The reliability and accuracy of self-reported cancer diagnoses in NHANES have been evaluated in previous studies, which suggest that there is generally good concordance between self-reported data and medical records for most common cancer types.

### Ascertainment of mortality

Mortality data for this study were obtained from the National Death Index (NDI) death certificate records provided by the NCHS, with the associated mortality file updated to December 31, 2019. The study outcomes included all-cause mortality and cause-specific mortality attributable to cancer and non-cancer causes, with causes of death based on the International Classification of Diseases (ICD). All-cause mortality was defined as deaths from any cause, encompassing cancers (C00-C97), cardiovascular disease (CVD) (I00-I09, I11, I13, I20-I51), cerebrovascular disease (I60-I69), respiratory disease (J10-J18, J40-J47), and other causes. Death due to malignancy was classified as cancer mortality (C00-C97) during the follow-up period. The duration from the baseline interview to the date of death or December 31, 2019, was calculated for each participant.

### Assessment of covariates

Covariates included demographic information, health behaviors, physical examination findings, and medical history. Demographic information was collected through self-administered NHANES questionnaires and encompassed basic details such as age, gender, race/ethnicity (non-Hispanic white, Hispanic, non-Hispanic black, and other), marital status, and the poverty-to-income ratio (PIR). Body mass index (BMI) was calculated as weight (in kilograms) divided by height squared (in meters) and classified as <25 (normal), 25.0-29.9 (overweight), or ≥ 30 kg/m² (obese). Smoking status was categorized into never-smokers, former smokers, and current smokers. Drinking status is divided into never-drinkers, former drinkers, and current drinkers. Hypertension was identified if blood pressure (BP) measurements were above 140/90 mm Hg or if patients were taking antihypertensive medication. Diagnosis of diabetes was based on laboratory measurements of fasting glucose and hemoglobin A1c, self-reported medication use, or a prior diagnosis by a healthcare provider. CVD was assessed through self-reported history and physical examinations. Chronic kidney disease (CKD) is identified by a glomerular filtration rate (eGFR) of less than 60 mL/min/1.73 m² or a urinary albumin-to-creatinine ratio of greater than 30 mg/g. The CKD-EPI formula of the Chronic Kidney Disease Epidemiology Collaboration was applied to calculate the eGFR.

### Statistical analysis

This study strictly followed the design characteristics of the NHANES stratified multi-stage probability sampling method, and composite sampling weights were used to correct selection bias and non-response bias. Grouping and analysis were based on the baseline NPAR quartiles (Q1≤ 12.70, 12.70< Q2≤ 14.40, 14.40< Q3 ≤ 16.17, Q4<16.17), continuous variables are expressed as mean ± standard deviation, and the Wilcoxon rank sum test was used for intergroup comparisons; categorical variables are described by frequency/percentage, and the chi-square test was used to test for differences between groups. The association between NPAR and mortality risk was explored in three stages using a Cox proportional hazards model: model 1 (unadjusted), model 2 (adjusted for age, gender, and race), and model 3 (further adjusted for additional factors including PIR, marital status, educational level, BMI, smoking status, alcohol consumption, diabetes, hypertension, and CVD. Survival curves were plotted using the Kaplan-Meier method, and the Log-rank test was used to assess differences between groups. A restricted cubic spline (RCS, 4 knots) was used to analyze the dose-response relationship between NPAR and mortality risk. Subgroup analyses included stratification variables such as age, gender, race, BMI, smoking status, alcohol consumption, hypertension, diabetes, CVD, and CKD. In addition, the mediating effect of renal function (quantified by eGFR) in the association between NPAR and mortality was further assessed. Finally, we performed receiver operating characteristic (ROC) curve analysis to evaluate the discriminatory ability of NPAR and the systemic immune-inflammation index (SII) in predicting all-cause, cancer-specific, and non-cancer mortality. The area under the curve (AUC) was calculated for each biomarker, and comparisons were made to assess their prognostic performance.

All analyses were performed using R 4.2.1 software, and a two-sided *P <*0.05 was considered statistically significant.

## Results

### Baseline characteristics of study participants

The study ultimately included 3,134 cancer survivors, stratified by NPAR quartile completion cohort (Q1-Q4). Baseline analysis revealed significant intergroup heterogeneity: the mean age (65.97 ± 13.40 vs 60.19 ± 13.99 years) and BMI (30.24 ± 7.34 vs 27.96 ± 5.32 kg/m²) of patients in the Q4 group (highest NPAR level) were significantly higher than those in the Q1 group (both *P <*0.001), which was accompanied by a higher burden of chronic diseases (hypertension 67.74% vs 53.24%; diabetes 30.51% vs 17.14%; CVD 25.09% vs 15.50%; CKD 26.02% vs 13.32%). It is worth noting that this group exhibits contradictory behavioral characteristics: there is a significant increase in the proportion of people living alone (37.24% vs. 29.00%, *P*=0.028) but a significant decrease in alcohol intake (60.36% of current drinkers vs. 70.92%, *P*=0.003). Survival outcome analysis showed that the all-cause mortality rate (32.80%), cancer-specific mortality rate (10.32%), and non-cancer mortality rate (22.48%) in the Q4 group were significantly higher than those in other groups (all *P <*0.001). See **Table 1** for details.

**Table 1:** Baseline characteristics of study participants by NPAR quartiles.

Characteristic	Overall N=17,551,655	Q1≤ 12.70 N=4,544,951	12.70<Q2 ≤ 14.40 N=4,776,283	14.40<Q3 ≤ 16.17 N=4,331,234	Q4>16.17 N=3,899,186	*P*-value
Age, years, mean (SD)	62.51 ± 14.32	60.19 ± 13.99	60.81 ± 14.01	63.69 ± 15.08	65.97 ± 13.40	<0.001
Gender						0.4
Male	1,483 (42.98%)	359 (43.35%)	351 (41.57%)	382 (41.09%)	391 (46.39%)	
Female	1,651 (57.02%)	427 (56.65%)	431 (58.43%)	400 (58.91%)	393 (53.61%)	
Race						0.2
Mexican American	197 (2.20%)	46 (1.89%)	47 (2.13%)	52 (2.55%)	52 (2.25%)	
Other Hispanic	162 (2.09%)	45 (2.57%)	51 (2.27%)	40 (1.88%)	26 (1.57%)	
Non-Hispanic white	2,230 (87.51%)	516 (85.71%)	570 (88.88%)	572 (87.82%)	572 (87.60%)	
Non-Hispanic black	411 (4.76%)	140 (6.58%)	85 (3.75%)	89 (3.83%)	97 (4.91%)	
Other race	134 (3.43%)	39 (3.25%)	29 (2.97%)	29 (3.92%)	37 (3.67%)	
Education level						0.2
Less than high school	653 (12.69%)	153 (12.83%)	150 (10.59%)	169 (13.11%)	181 (14.65%)	
High school or GED	1,677 (53.96%)	423 (51.67%)	419 (53.84%)	415 (54.08%)	420 (56.63%)	
Above high school	804 (33.35%)	210 (35.51%)	213 (35.57%)	198 (32.80%)	183 (28.72%)	
Marital status						0.028
Married or living with a partner	1,904 (66.04%)	509 (71.00%)	481 (66.37%)	469 (63.44%)	445 (62.76%)	
Living alone	1,230 (33.96%)	277 (29.00%)	301 (33.63%)	313 (36.56%)	339 (37.24%)	
PIR	3.30 ± 1.56	3.44 ± 1.56	3.38 ± 1.59	3.21 ± 1.54	3.12 ± 1.54	0.004
BMI, kg/m2, mean (SD)	28.93 ± 6.45	27.96 ± 5.32	28.34 ± 6.01	29.42 ± 6.89	30.24 ± 7.34	<0.001
Alcohol consumption						0.003
Never drinkers	383 (9.52%)	103 (10.21%)	95 (8.92%)	88 (9.03%)	97 (9.99%)	
Former drinkers	746 (23.37%)	170 (18.87%)	165 (19.79%)	186 (26.40%)	225 (29.65%)	
Current drinkers	2,005 (67.11%)	513 (70.92%)	522 (71.29%)	508 (64.57%)	462 (60.36%)	
Smoking status						0.3
Never smoker	1,388 (45.22%)	388 (48.46%)	340 (43.20%)	344 (47.24%)	316 (41.69%)	
Former smoker	1,260 (38.70%)	286 (37.31%)	315 (39.78%)	332 (38.44%)	327 (39.29%)	
Current smoker	486 (16.07%)	112 (14.24%)	127 (17.01%)	106 (14.31%)	141 (19.02%)	
Medical history						
Hypertension	2,012 (57.95%)	487 (54.04%)	473 (53.24%)	509 (58.44%)	543 (67.74%)	<0.001
Diabetes	838 (22.18%)	172 (17.14%)	191 (18.55%)	222 (23.98%)	253 (30.51%)	<0.001
CVD	775 (19.48%)	150 (15.50%)	189 (18.12%)	202 (20.10%)	234 (25.09%)	0.002
CKD	734 (18.33%)	141 (13.32%)	156 (15.68%)	198 (19.60%)	239 (26.02%)	<0.001
All-cause mortality, n (%)						<0.001
Alive	2,216 (79.13%)	620 (84.96%)	593 (83.75%)	539 (78.67%)	464 (67.20%)	
Death	918 (20.87%)	166 (15.04%)	189 (16.25%)	243 (21.33%)	320 (32.80%)	
Cancer mortality, n (%)						<0.001
Alive	2,853 (93.54%)	722 (94.27%)	726 (95.39%)	718 (94.23%)	687 (89.68%)	
Death	281 (6.46%)	64 (5.73%)	56 (4.61%)	64 (5.77%)	97 (10.32%)	
Non-cancer mortality, n (%)						<0.001
Alive	2,497 (85.59%)	684 (90.69%)	649 (88.36%)	603 (84.44%)	561 (77.52%)	
Death	637 (14.41%)	102 (9.31%)	133 (11.64%)	179 (15.56%)	223 (22.48%)	

NPAR, neutrophil percentage-to-albumin ratio; PIR, poverty income ratio; BMI, body mass index; CVD, cardiovascular disease; CKD, Chronic kidney disease.

### Relationship between NPAR and mortality

During the median follow-up of 7.24 years, a total of 918 deaths were recorded, including 281 cancer-related deaths (30.6%) and 637 non-cancer-related deaths (69.4%). Stratified analysis showed a significant time-dose effect of NPAR level on the risk of death: the cumulative incidence of all-cause death (320 cases), cancer-related death (97 cases), and non-cancer-related death (223 cases) in the Q4 group (highest NPAR) was significantly higher than that in other groups (see **Table 1** for details).

The continuous variable model showed that for every one-unit increase in NPAR, the risk of all-cause mortality increased by 10%–15% (models 1–3: HR=1.10–1.15, all *P <*0.001), and the risk of non-cancer-related mortality increased by 12%–17% (HR=1.12–1.17, all *P <*0.001). It is worth noting that in the basic model (model 1: HR=1.10, 95%CI 1.04-1.17, *P* =0.001) and the population school correction model (model 2: HR=1.07, 1.01-1.14, *P* =0.025), NPAR remained a significant predictor of cancer-related mortality risk in the population-adjusted model (model 2: HR=1.07, 1.01-1.14, *P* =0.025), but the association weakened to borderline significance in the fully adjusted model (model 3) (HR=1.06, 1.00-1.13, *P* =0.054) (**Table 2**).

**Table 2:** Cox proportional hazards regression analysis of NPAR and mortality.

Exposure	Model 1 HR (95% CI) *P*-value	Model 2 HR (95% CI) *P*-value	Model 3 HR (95% CI) *P*-value
All-cause mortality			
NPAR (continuous)	1.15 (1.12, 1.19) <0.001	1.11 (1.08, 1.14) <0.001	1.10(1.07, 1.13) <0.001
NPAR quartile			
Quartile 1	Reference	Reference	Reference
Quartile 2	1.13 (0.90, 1.41) 0.309	1.10 (0.89, 1.35) 0.374	1.05 (0.85, 1.29) 0.632
Quartile 3	1.54 (1.20, 1.97) <0.001	1.14 (0.92, 1.41) 0.243	1.10 (0.89, 1.36) 0.370
Quartile 4	2.81 (2.25, 3.51) <0.001	2.04 (1.68, 2.49) <0.001	1.89 (1.55, 2.31) <0.001
P for trend	<0.001	<0.001	<0.001
Cancer mortality			
NPAR (continuous)	1.10 (1.04, 1.17) 0.001	1.07 (1.01, 1.14) 0.025	1.06 (1.00, 1.13) 0.054
NPAR quartile			
Quartile 1	Reference	Reference	Reference
Quartile 2	0.83 (0.55,1.25) 0.372	0.82 (0.55, 1.22) 0.327	0.81 (0.54, 1.21) 0.303
Quartile 3	1.08 (0.72, 1.62) 0.706	0.91 (0.60, 1.40) 0.674	0.88 (0.57, 1.36) 0.564
Quartile 4	2.23 (1.59, 3.13) <0.001	1.78 (1.27, 2.48) <0.001	1.67 (1.18, 2.35) 0.003
P for trend	<0.001	0.001	0.004
Non-cancer mortality			
NPAR (continuous)	1.17 (1.13, 1.22) <0.001	1.12 (1.08, 1.17) <0.001	1.12 (1.08, 1.16) <0.001
NPAR quartile			
Quartile 1	Reference	Reference	Reference
Quartile 2	1.31 (1.01, 1.70) 0.045	1.28 (1.00, 1.64) 0.049	1.20 (0.94, 1.53) 0.144
Quartile 3	1.82 (1.34, 2.48) <0.001	1.28 (0.98, 1.62) 0.074	1.22 (0.95, 1.55) 0.116
Quartile 4	3.16 (2.39, 4.18) <0.001	2.22 (1.72, 2.85) <0.001	2.04 (1.61, 2.58) <0.001
P for trend	<0.001	<0.001	<0.001

Model 1: No covariates were adjusted.

Model 2: Age, gender, and race were adjusted.

Model 3: Age, gender, race, PIR, marital status, educational level, BMI, smoking status, alcohol consumption, diabetes, hypertension, and CVD were adjusted.

NPAR, neutrophil percentage-to-albumin ratio; PIR, poverty income ratio; BMI, body mass index; CVD, cardiovascular disease; HR, hazard ratio; CI, confidence interval.

From a clinical significance perspective, each unit increase in NPAR is associated with a quantifiable increase in risk that holds clear clinical reference value. In the fully adjusted model (Model 3), an increase of 1 unit in NPAR is directly associated with a 10% increase in all-cause mortality risk (HR=1.10) and a 12% increase in non-cancer mortality risk (HR=1.12). This suggests that even a small rise in NPAR (for example, from 10 to 11) may lead to a quantifiable adverse change in the mortality risk of cancer survivors. Furthermore, the association between NPAR and cancer-specific mortality risk (HR=1.06, P=0.054), while approaching statistical significance, indicates a potential link between NPAR levels and tumor-related prognosis.

When NPAR was grouped into quartiles, the gradient effect of mortality risk was more significant: compared with Q1, the risk of all-cause mortality in Q4 increased by 89% (model 3 HR=1.89, 95%CI 1.55-2.31), and the risk of non-cancer-related mortality increased by 104% (HR=2.04, 1.61-2.58) (both *P <*0.001). The risk of cancer-related death decreased but still maintained an independent risk increase of 67% (Q4 vs Q1: HR=1.67, 1.18-2.35, *P* =0.003) (**Table 2**).

### Survival outcomes by NPAR levels

**Figure 2:**
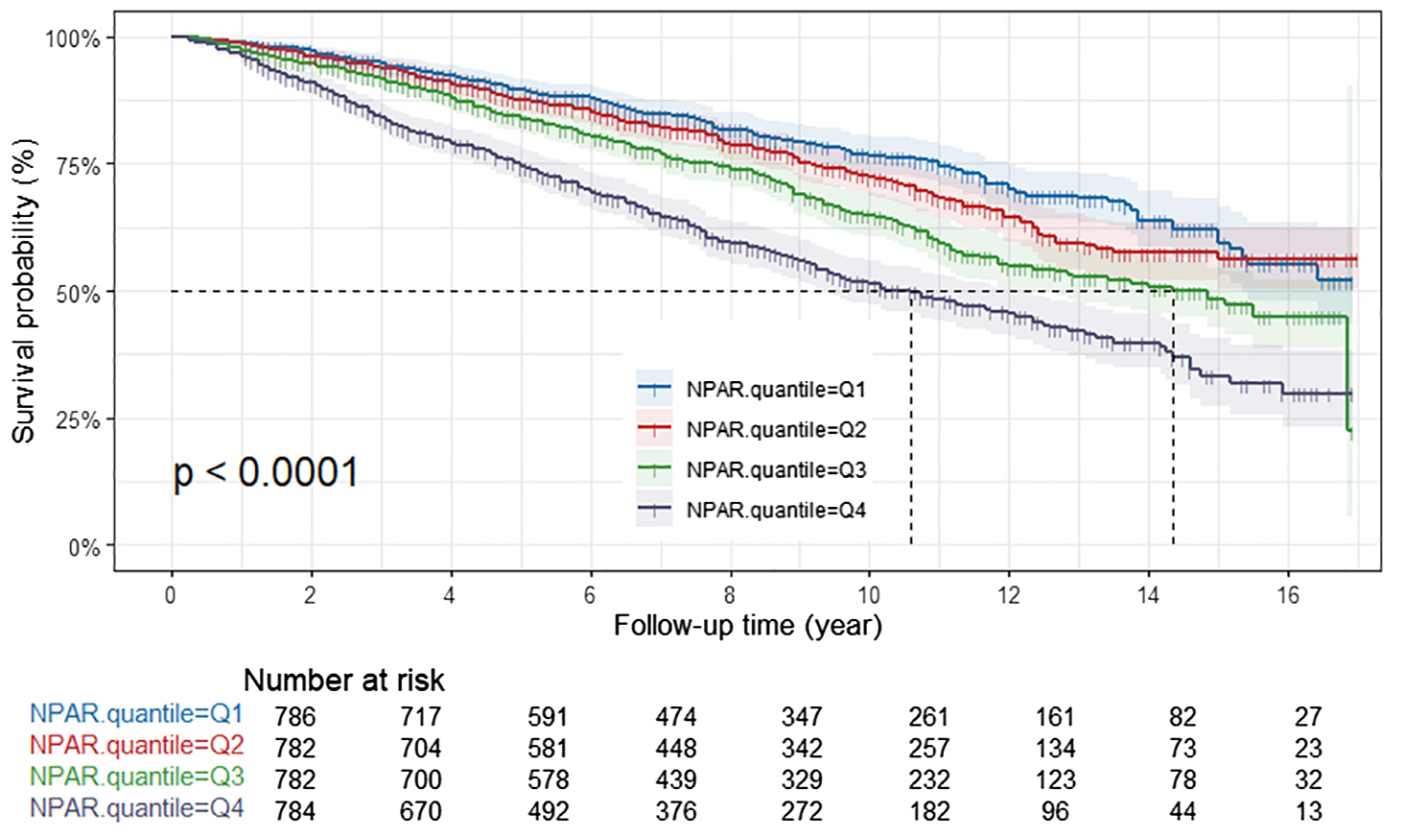
Kaplan-Meier curves for all-cause mortality by NPAR quartiles. NPAR, neutrophil percentage-to-albumin ratio.

**Figure 3:**
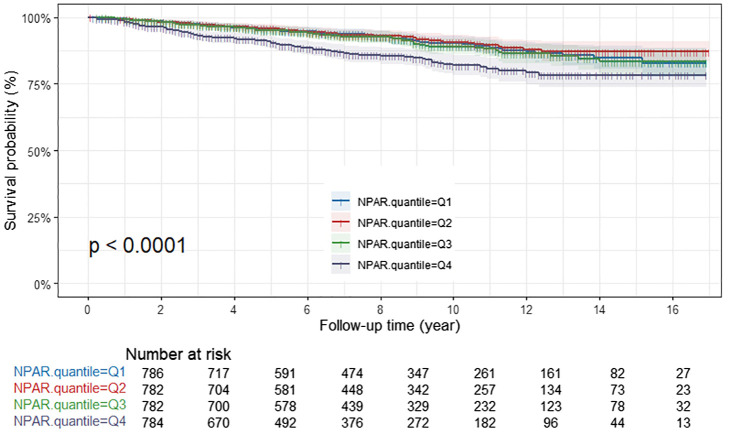
Kaplan-Meier curves for cancer-specific mortality by NPAR quartiles. NPAR, neutrophil percentage-to-albumin ratio.

**Figure 4:**
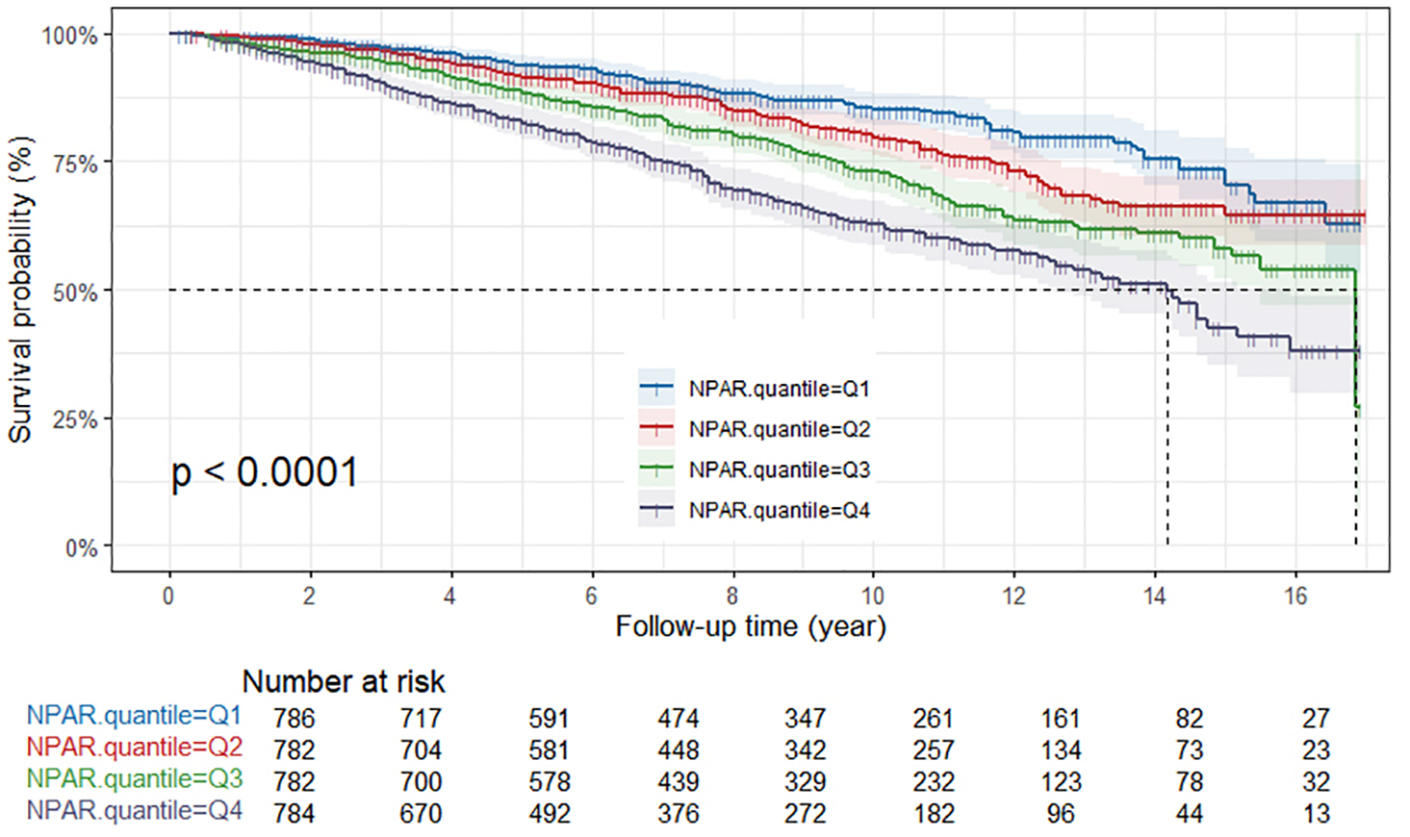
Kaplan-Meier curves for non-cancer-specific mortality by NPAR quartiles. NPAR, neutrophil percentage-to-albumin ratio.

In the Kaplan-Meier survival analysis, all-cause survival rates for patients tended to decline with increasing NPAR, and this observation was statistically significant (P<0.001) (**Figure 2**). Patients in the Q4 group exhibited the poorest cancer-specific survival rates, with the difference being statistically significant (*P*<0.001) (**Figure 3**). Furthermore, non-cancer-specific survival rates also decreased with rising NPAR levels, with a statistically significant difference observed (P<0.001) (**Figure 4**).

### Nonlinear association between NPAR and mortality

**Figure 5:**
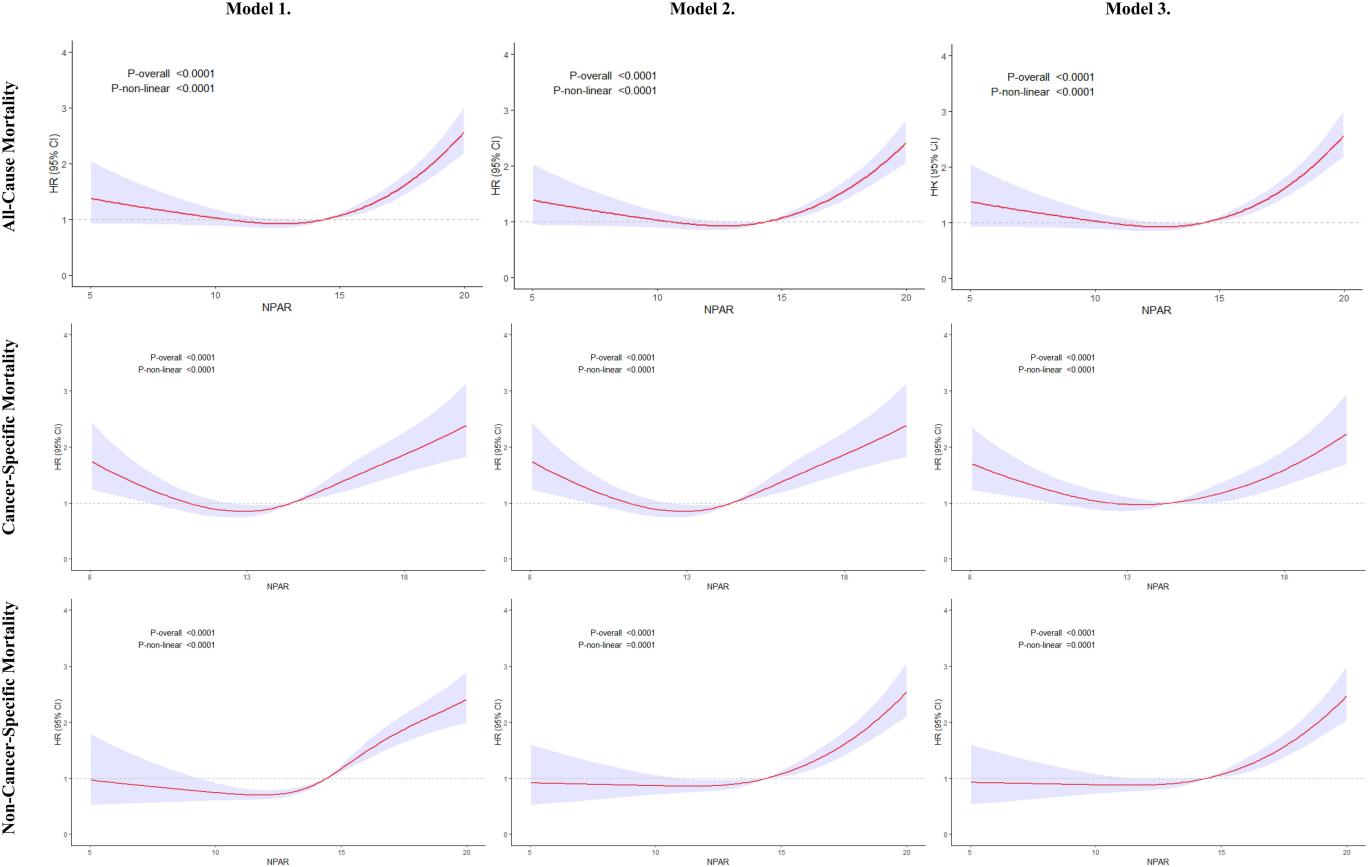
Restricted cubic spline analysis of the association between NPAR and mortality in Cancer Survivors. Model 1 represents unadjusted covariates; Model 2 is adjusted for age, gender, and race; and Model 3 is adjusted for age, gender, race, PIR, marital status, educational level, BMI, smoking status, alcohol consumption, diabetes, hypertension, and CVD. The solid line and purple area represent estimates and their corresponding 95% confidence intervals (CIs), respectively. NPAR, neutrophil percentage-to-albumin ratio; PIR, ratio of income to poverty; BMI, body mass index; CVD, cardiovascular disease; HR, hazard ratio; CI, confidence interval.

The results of restricted cubic splines (RCS) analysis demonstrate that the relationship between NPAR and mortality risk is not merely linear. When NPAR is below 12.84, each unit increase in NPAR is associated with a gradual decline in all-cause mortality risk (HR=0.92, 95% CI: 0.88-0.97, *P*<0.001). However, once NPAR surpasses this threshold of 12.84, each unit increase in NPAR leads to a dramatic 16% rise in all-cause mortality risk (HR=1.16, 95% CI: 1.13-1.19, *P <*0.001). This nonlinear characteristic underscores the importance of closely monitoring cancer survivors with NPAR levels of 12.84 or higher in clinical practice. In this population, the risk effect associated with each unit increase in NPAR (HR=1.16) is notably greater than the overall linear estimate (HR=1.10), necessitating more proactive interventions to address inflammatory and nutritional status in order to reduce mortality risk (**Figure 5**, **Table 3**).

**Table 3:** Threshold effect analysis of NPAR index on all-cause mortality in cancer survivors.

All-cause mortality	HR (95% CI) *P* value
Fitting by the standard linear model	1.10 (1.07, 1.12) <0.001
Fitting by the two-piecewise linear model	
Inflection point	12.84
NPAR index<12.84	0.92 (0.88, 0.97) <0.001
NPAR index ≥ 12.84	1.16 (1.13, 1.19) <0.001
P for Log-likelihood ratio	<0.001

The model was adjusted for age, gender, race, education, marital level, PIR, BMI, drinker, smoker, diabetes, hypertension, and CVD. NPAR, neutrophil percentage-to-albumin ratio; PIR, poverty income ratio; BMI, body mass index; CVD, cardiovascular disease; HR, hazard ratio; CI, confidence interval.

### Subgroup associations of NPAR With mortality

**Figure 6:**
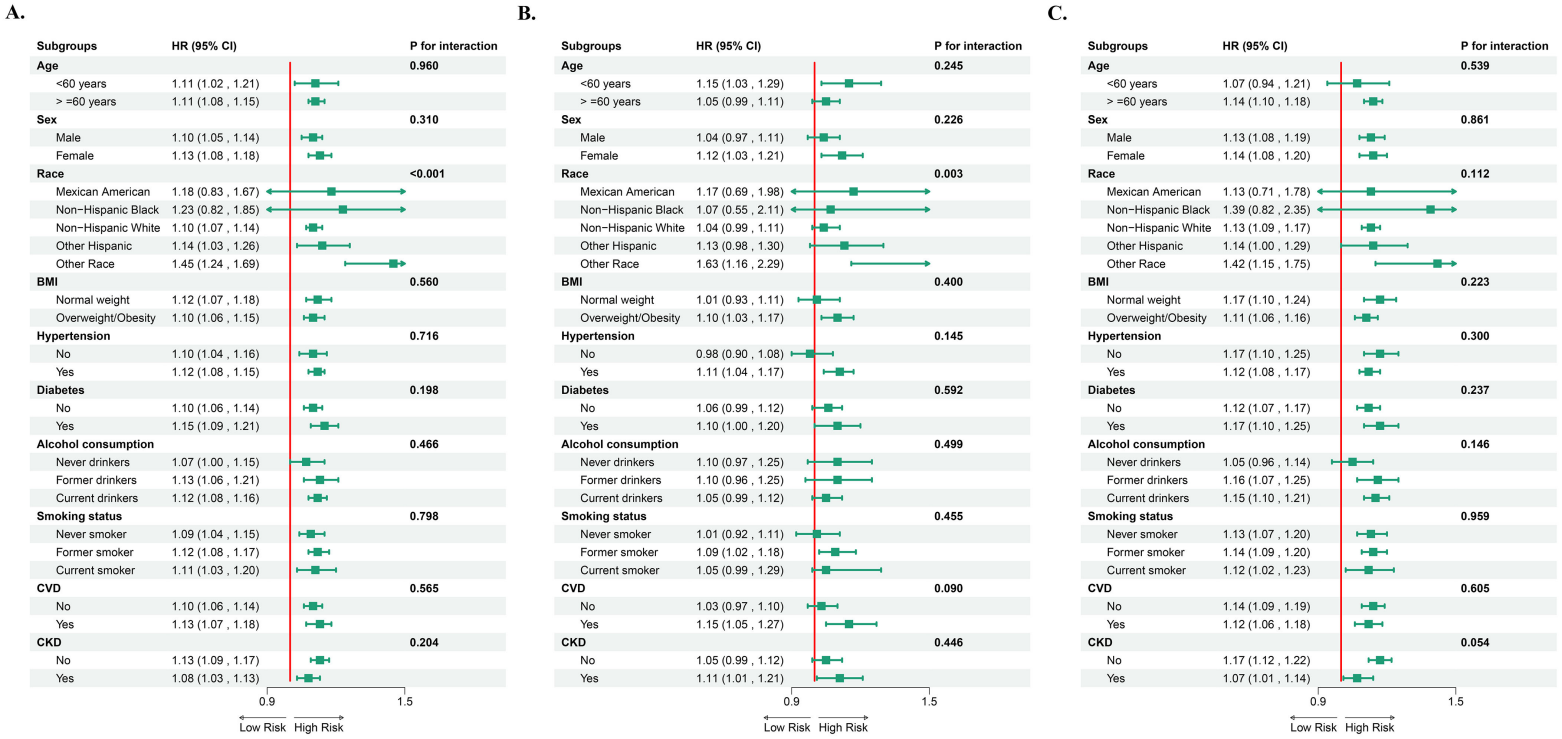
Subgroup analysis between NPAR and all-cause mortality **(A)**, cancer-specific mortality **(B)**, and non-cancer-specific mortality **(C)**. NPAR, neutrophil percentage-to-albumin ratio; BMI, body mass index; CVD, cardiovascular disease; CKD, Chronic kidney disease; HR, hazard ratio; CI, confidence interval.

Subgroup analyses revealed no significant heterogeneity in the association of NPAR with the risk of all-cause and idiosyncratic mortality (both interaction *P* > 0.05). However, specific clinical subgroups showed a more significant risk gradient: for all-cause mortality, females (HR=1.13 vs. males HR=1.10), normal weight individuals (HR=1.12 vs. overweight/obese HR=1.10), those with combined hypertension (HR=1.12), diabetes mellitus (HR=1.15), or CVD (HR=1.13), as well as non-CKD patients exhibited a more pronounced increase in risk. In contrast, for cancer-specific mortality, individuals aged <60 years (HR=1.15 vs. ≥60 years HR=1.05), females (HR=1.12), overweight/obese individuals (HR=1.10), and those with comorbid hypertension (HR=1.11), diabetes (HR=1.10), CVD (HR=1.15), or CKD (HR=1.11) displayed a stronger NPAR-mortality risk association (**Figure 6**).

### Mediating role of renal function

**Figure 7:**
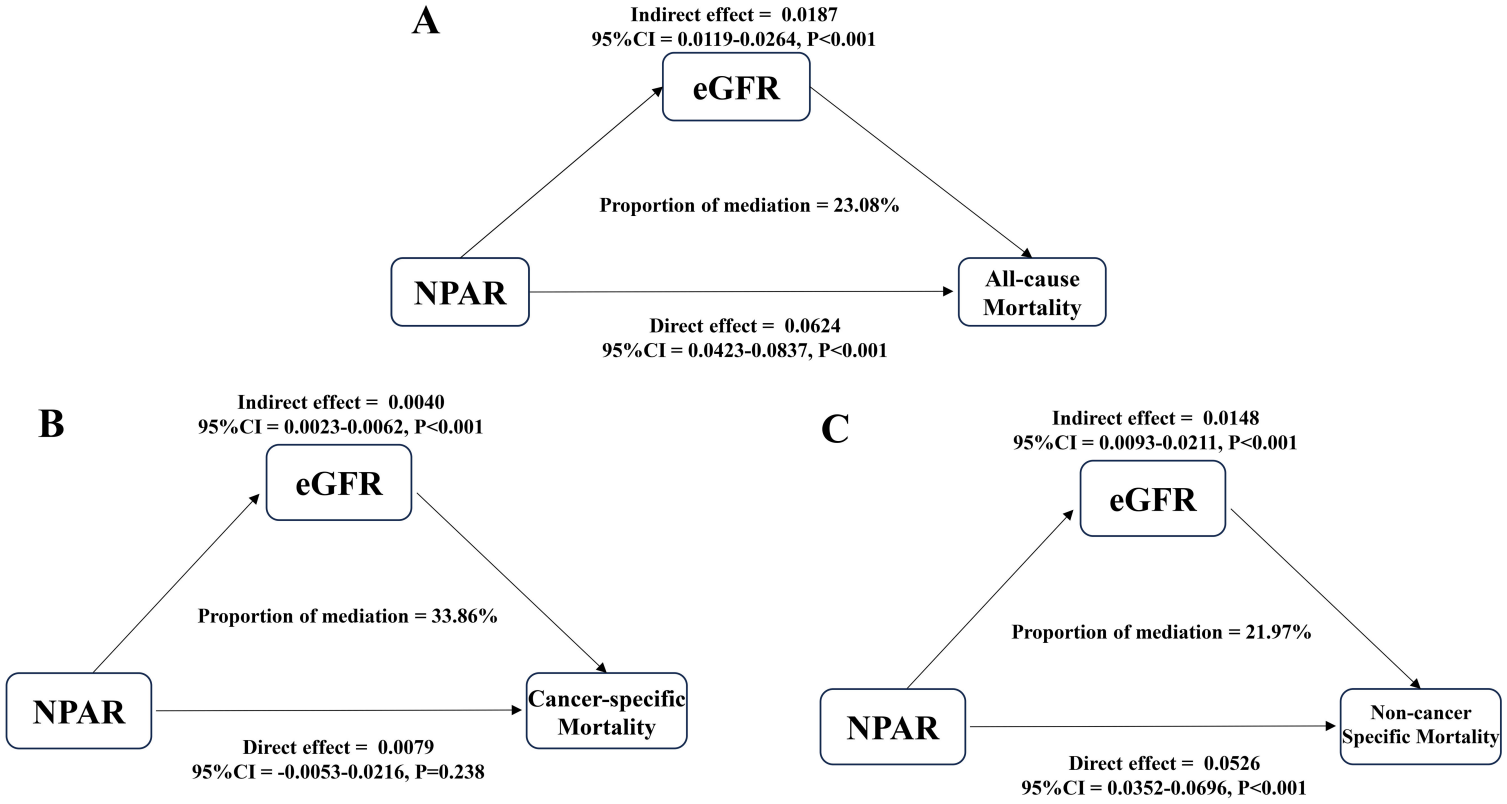
Analysis of the mediation by eGFR of the associations of NPAR with all-cause mortality **(A)**, cancer-specific mortality **(B)**, and non-cancer-specific mortality **(C)** in cancer survivors. NPAR, neutrophil percentage-to-albumin ratio; eGFR, glomerular filtration rate.

This study innovatively introduced the causal mediation analysis framework, which revealed for the first time the key mediating role of renal function (eGFR) in the association between NPAR and mortality. For all-cause mortality (**Figure 7A**), NPAR exerted an indirect effect on all-cause mortality through eGFR, with an indirect effect value of 0.0187 (95% CI: 0.0119-0.0264, *P*<0.001). The direct effect of NPAR on all-cause mortality was 0.0624 (95% CI: 0.0423 - 0.0837, *P*<0.001), and the proportion of mediation accounted for by eGFR was 23.08%. Regarding cancer-specific mortality (**Figure 7B**), the indirect effect of NPAR on cancer-specific mortality via eGFR was 0.0040 (95% CI: 0.0023 - 0.0062, *P*<0.001). However, the direct effect of NPAR on cancer-specific mortality was 0.0079 (95% CI: -0.0053 - 0.0216, *P*=0.238), and the proportion of mediation by eGFR was 33.86%. Notably, no significant direct effect of NPAR on cancer-specific mortality was observed. For non-cancer-specific mortality (**Figure 7C**), NPAR had an indirect effect on non-cancer-specific mortality through eGFR, with a value of 0.0148 (95% CI: 0.0093 - 0.0211, *P*<0.001). The direct effect of NPAR on non-cancer-specific mortality was 0.0526 (95% CI: 0.0352 - 0.0696, *P*<0.001), and the proportion of mediation by eGFR was 21.97%.

### Discriminatory performance of NPAR and SII

**Figure 8:**
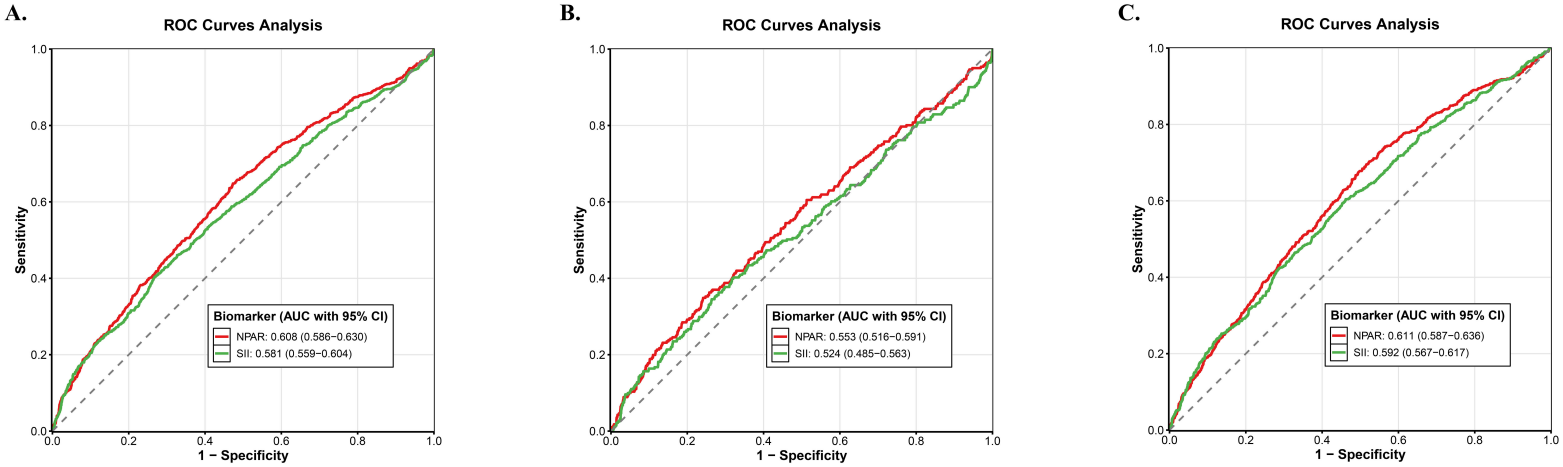
ROC curve analysis of the predictive value of NPAR and SII for all-cause mortality **(A)**, cancer-specific mortality **(B)** and non-cancer mortality **(C)**.

ROC curve analyses were performed to evaluate the discriminatory ability of NPAR and SII in predicting all-cause mortality, cancer-specific mortality and non-cancer. For all-cause mortality (**Figure 8A**), the AUC for NPAR was 0.608 (95% CI: 0.586–0.630), while the AUC for SII was 0.581 (95% CI: 0.559–0.604). When assessing cancer-specific mortality (**Figure 8B**), NPAR exhibited an AUC of 0.553 (95% CI: 0.516–0.591) and SII an AUC of 0.524 (95% CI: 0.485–0.563). In the context of non-cancer-specific mortality prediction (**Figure 8C**), the AUC for NPAR was 0.611 (95% CI: 0.587–0.636) and for SII it was 0.592 (95% CI: 0.567–0.617). Overall, NPAR demonstrated marginally superior discriminatory capacity compared with SII across all the mortality endpoints examined.

### External validation analyses

To further validate the relationship between NPAR and cancer patient prognosis, a real-world data analysis was conducted at Longyan First Hospital with ethical approval obtained (Ethics Approval Number: LYREC2024-k028-01). A total of 1,194 patients diagnosed with cancer were enrolled between January 2016 and December 2018. Among these, 59 lacked neutrophil count or serum albumin data, 126 had missing follow-up data, and 24 lacked covariates. Ultimately, 985 participants were included in the analysis. **Supplementary Table 1** presents baseline characteristics of study participants stratified by NPAR.

Consistently, participants with higher NPAR levels exhibited increased all-cause mortality, cancer-specific mortality, and non-cancer-specific mortality (all *P*<0.001) (**Supplementary Table S2**). After adjusting for covariates including age, sex, BMI, smoking status, alcohol consumption, hypertension, diabetes, and heart disease, the positive association remained statistically significant (all *P*<0.001). When NPAR was grouped into quartiles, compared with Q1, Q4 was associated with a 163% increased risk of all-cause mortality (Model 3 HR=2.63, 95% CI 1.87-3.71), cancer-related mortality risk increased by 224% (HR=3.24, 1.76-5.98), and non-cancer-related mortality risk increased by 84% (HR=1.84, 1.25-2.70).

Furthermore, Kaplan-Meier survival analysis of the external validation cohort revealed that overall survival, cancer-specific survival, and non-cancer-specific survival rates all exhibited a declining trend with increasing NPAR, patients in the Q4 group demonstrated the lowest survival rates, with the difference being statistically significant (all *P*<0.001) (**Supplementary Figure S1-3**).

## Discussion

This study utilized data from NHANES and revealed for the first time that NPAR is an independent predictor of all-cause and cancer-specific mortality risk in cancer survivors. The core findings showed that for every 1-unit increase in NPAR, the risk of all-cause mortality increased by 10%, the risk of cancer-related mortality increased by 6%, and the risk of non-cancer mortality increased by 12%. This dose-dependent association remained robust in multivariate adjusted models. RCS analysis further revealed a nonlinear relationship between NPAR and mortality risk, with a steep inflection point in the risk of all-cause mortality when NPAR exceeded 12.84. Finally, mediation analysis clarified that renal function impairment is a key pathway through which NPAR affects prognosis: 23.08% of the risk of all-cause mortality mediated by NPAR is driven by a decline in eGFR, and 21.97% of the risk of non-cancer mortality is attributed to worsening renal function.

Neutrophils represent a significant component of the innate immune system, with their hematopoietic stem cells generated through evolutionary processes. In the tumor microenvironment, neutrophils are classified as tumor-associated neutrophils (21). Numerous studies have demonstrated that neutrophils exhibit plasticity within the tumor host; they can be categorized into anti-tumor (N1) and pro-tumor (N2) phenotypes based on their roles in tumor dynamics. Neutrophils of the N1 phenotype may exert anti-tumor effects via reactive oxygen species (ROS)-associated pathways, while pro-tumor mechanisms of N2 phenotype neutrophils may involve neutrophil elastase (NE) and matrix metalloproteinases (22–24). Albumin can bind to pro-inflammatory cytokines, such as IL-6, and neutralize lipopolysaccharides (LPS), thereby preventing the conversion of neutrophils to the N2 phenotype. *In vitro* studies (25) have demonstrated that albumin can inhibit the formation of NETs (neutrophil extracellular traps) by N2 neutrophils, thereby reducing their protective effect on tumor cells. A decrease in albumin levels, which is commonly observed in high NPAR states, increases the availability of free IL-6, which in turn induces N2 polarization. This creates a vicious cycle: N2 neutrophils secrete more IL-6, further inhibiting hepatic albumin synthesis, leading to even higher NPAR levels and ultimately resulting in poorer prognosis (25). The N1/N2 ratio has also been suggested as a prognostic factor in hepatocellular carcinoma, where a higher N1/N2 ratio in the tumor microenvironment correlates positively with prognosis. Conversely, a high N1/N2 ratio in peritumoral tissues has been associated with poorer prognostic outcomes (26). Additionally, tumor-associated neutrophils may be influenced by metabolic processes, leading to increased expression of hypoxia-inducible factor 1α (HIF-1α), which promotes angiogenesis, glycolysis, and gene upregulation, ultimately advancing tumor progression (27–30). Furthermore, past studies have demonstrated that neutrophil infiltration is regulated by chemokine pathways such as CXCL/CXCR2, which are associated with either improved or worsened outcomes in different cancer types, highlighting their complex roles in cancer progression (31, 32).

Neutrophils and lymphocytes serve as critical and easily accessible markers of inflammation; the significance of the neutrophil-to-lymphocyte ratio in prognosing various diseases has garnered considerable interest. Elevations in this ratio may constitute an important risk factor for increased cardiovascular mortality among cancer survivors (33). Importantly, inflammation among cancer survivors is routinely correlated with nutritional status, and recent studies indicate that cancer patients exhibit a heightened risk of malnutrition, with their nutritional and immune statuses serving as significant predictors of tumor development and outcomes (12, 33, 34). In the context of disease prediction, albumin levels are closely correlated with inflammation and can serve as a marker for clinical stability when assessing nutritional status (35, 36). Albumin and globulin represent the primary components of serum proteins, with numerous studies confirming their efficacy as indicators of nutritional and inflammatory status in cancer progression. Their prognostic significance has been established across multiple cancers, including non-small cell lung cancer, gastric cancer, colorectal cancer, and breast cancer (37–40).

Several comprehensive indicators have been used to assess inflammation and nutritional status in cancer patients, including hemoglobin, albumin, lymphocytes, and platelets (HALP score) (41), neutrophil-to-lymphocyte ratio (NLR), platelet-to-lymphocyte ratio (PLR) (6), prognostic nutritional index (PNI) (8), and the systemic immune-inflammation index (SII) (7). Compared to these indices, NPAR has distinct advantages as it combines a direct measure of innate immune activity (neutrophil percentage) with reliable indicators of nutritional and inflammatory status (albumin). While NLR and PLR reflect immune imbalance, and PNI and HALP include nutritional components, NPAR provides a more comprehensive assessment of systemic inflammation and catabolic status, which may explain its robust predictive performance in our cohort.

The NPAR integrates inflammatory and nutritional factors to provide a comprehensive assessment of an individual’s inflammatory and nutritional status, and it was first introduced in 2016 by Bernard et al., demonstrating its potential predictive value in cancer (13). Numerous studies have supported the predictive validity of NPAR in various chronic diseases, revealing a positive correlation between high NPAR and both all-cause mortality and cardiovascular mortality in patients with hypertension and diabetes (42, 43). Additionally, elevated NPAR has been implicated as a risk factor for stroke (44). In the realm of tumor-specific survival, a growing body of research has established NPAR’s predictive value in patients with bladder cancer undergoing surgery after neoadjuvant chemotherapy, linking higher NPAR to lower overall tumor survival. Conversely, in oral squamous cell carcinoma, high NPAR has been associated with poorer overall survival and disease-free survival in patients (14, 45). A multicenter cohort study has shown high NPAR to be independently associated with all-cause mortality in individuals with cancer (46), aligning with our findings that indicate that higher NPAR correlates with poorer prognoses among cancer survivors. In terms of all-cause mortality, these results suggest that higher NPAR may be associated with an increased mortality risk, and we have identified possible inflection points. Furthermore, our additional analyses concerning cancer-specific and non-cancer mortality led to similar conclusions, indicating that elevated NPAR is associated with an increased risk of both cancer-specific and non-cancer-related deaths.

A novel finding of this study is the mediating role of renal function in the association between NPAR and mortality. Renal impairment may exacerbate systemic inflammation and malnutrition through several mechanisms. The interaction mechanism between renal dysfunction and systemic inflammation, as well as malnutrition, suggests that renal impairment (evidenced by decreased estimated glomerular filtration rate) is not an isolated pathological state but forms a “vicious cycle” through various pathways involving systemic inflammation and malnutrition, thereby exacerbating the mortality risk among cancer survivors. From an inflammatory amplification perspective, the kidneys typically maintain inflammatory homeostasis by clearing circulating inflammatory mediators (such as tumor necrosis factor-alpha and interleukin-6), metabolizing reactive oxygen species (ROS), and inhibiting the overactivation of neutrophils. When estimated GFR (eGFR) declines, this “inflammatory clearance capacity” is severely impaired: accumulated inflammatory factors (such as interleukin-6) further activate neutrophils and promote the formation of neutrophil extracellular traps (NETs), a process associated with elevated NPAR in this study. Activated NETs exacerbate Toll-like receptor 4/NF-κB signaling in renal tubules, triggering a more intense local renal inflammatory response and establishing a positive feedback loop of “renal inflammation → systemic inflammation → renal injury” (47, 48).Second, hypoalbuminemia creates a vicious cycle by inhibiting vascular endothelial growth factor (VEGF) signaling and mTORC1-mediated autophagy repair mechanisms, a process that has been validated in mouse models (49, 50). Notably, the S100A8/A9 proteins secreted by neutrophils establish positive feedback with hypoalbuminemia through bidirectional regulation — the former inhibits HNF-4α, a key factor in liver albumin synthesis, while the latter promotes the further release of NETs by upregulating HMGB1 (51). Importantly, the downregulation of Klotho expression in renal tubules not only aggravates phosphate retention and vascular calcification but also maintains the stemness of tumor cells through the Wnt/β-catenin pathway (52), suggesting that the imbalance of the Klotho/FGF23 axis may be a central mediator in the “renal-cancer dialogue.” Future studies should combine single-cell sequencing to elucidate cell interactions in the renal microenvironment and develop targeted intervention strategies aimed at clearing NETs or regulating the albumin-VEGF axis.

However, the underlying mechanisms through which NPAR contributes to increased mortality remain incompletely understood. Current studies have predominantly focused on inflammatory mechanisms, nutritional status, and immune dysregulation in the context of tumorigenesis, while the role of renal function has been relatively underexplored. Importantly, our mediation analysis identifies renal impairment (reflected by reduced eGFR) as a critical intermediary pathway linking NPAR to poor outcomes. Neutrophils may exert pro-tumorigenic effects through the secretion of cytokines and chemokines, the formation of neutrophil extracellular traps (NETs), and intercellular interactions, thereby promoting tumor angiogenesis, cancer cell proliferation, and metastasis (11). Notably, impaired renal clearance capacity due to decreased eGFR exacerbates systemic inflammation by failing to effectively remove pro-inflammatory cytokines (e.g., IL-6, TNF-α), which in turn further activates neutrophils and promotes NET formation. Meanwhile, albumin—a key component of NPAR—is not only a marker of nutritional status but also possesses anti-inflammatory properties. It has been demonstrated that serum albumin can inhibit NET formation *in vitro* by neutralizing activators such as lipopolysaccharide (LPS) (25). In the setting of renal dysfunction, hypoalbuminemia may intensify this vicious cycle by impairing antioxidant and endotoxin-neutralizing capacities, thereby amplifying neutrophil-mediated inflammation and organ damage. Therefore, renal dysfunction serves as both a contributor to and a consequence of imbalanced inflammatory-nutritional status, forming a self-reinforcing cycle that ultimately increases mortality risk. Nevertheless, the precise mechanisms underlying NPAR’s predictive value, particularly its interplay with renal pathways, warrant further investigation in future studies.

It is important to acknowledge the potential heterogeneity across different cancer types in our study. The biological behavior, tumor microenvironment, and treatment modalities vary substantially among malignancies (e.g., solid tumors vs. hematologic cancers, or hormone-driven vs. inflammation-driven cancers). These differences could undoubtedly influence the relationship between systemic inflammation/nutrition and patient survival. This study, by design, focused on the general population of cancer survivors to provide an overarching insight into the NPAR-mortality link. This approach has the advantage of identifying a biomarker with broad applicability but may obscure its cancer-specific performance. Future research is imperative to validate and calibrate the prognostic value of NPAR within homogenous cohorts of specific cancer types to guide precise clinical application.

This study does have some limitations. First, it is a retrospective study, and its design does not completely eliminate the possibility of selection bias. Although various confounding factors have been adjusted for, residual confounding (such as unmeasured lifestyle factors and genetic background) might still affect the results. Second, due to the observational design, the possibility of reverse causation cannot be fully excluded (for instance, elevated NPAR before death may reflect end-stage pathological states). Third, conclusions drawn from the NHANES U.S. population may not be directly applicable to other ethnic groups or healthcare systems, requiring further validation for external applicability. Finally, although the NHANES is nationally representative and features a rigorous research design, it lacks internal validation from independent clinical cohorts. Future studies should validate our findings in prospective, multi-center cohorts to enhance clinical translatability.

## Conclusion

This study identifies the NPAR as a novel prognostic biomarker for mortality in cancer survivors. Elevated NPAR independently correlates with increased risks of all-cause, cancer-specific, and non-cancer mortality. Mediation analysis identifies renal dysfunction as a key pathway connecting NPAR to adverse outcomes, highlighting the interplay between systemic inflammation, nutrition, and kidney health. These findings support NPAR’s clinical utility for risk stratification and advocate for interventions targeting the inflammation-nutrition-kidney nexus to improve survival.

## Data Availability

The raw data supporting the conclusions of this article will be made available by the authors, without undue reservation.
