# Supplementary material for: Renal function mediates the association between neutrophil percentage-to-albumin ratio and survival in cancer survivors: a large cross-sectional study
Source: Front Oncol. 2026 Jan 14;15:1620484. doi: 10.3389/fonc.2025.1620484 (PMC12847009; doi:10.3389/fonc.2025.1620484)
Supplement: Supplementary file 1 [file DataSheet1.pdf]

Table S1. Baseline characteristics of study participants by NPAR quartiles from external validation cohort.

Characteristic	Overall n = 985	Q1 ≤ 7.90 n = 248	7.90< Q2 ≤ 10.10 n = 244	10.1< Q3 ≤ 13.48 n = 246	Q4 > 13.48 n = 247	*P*-value
**Age, years, mean (SD)**	63.80 ± 12.60	63.53 ± 11.82	63.97 ± 12.38	63.78 ± 13.50	63.92 ± 12.71	0.980
**Gender, n (%)**						<0.001
Male	371 (37.66%)	116 (46.77%)	99 (40.57%)	71 (28.86%)	85 (34.41%)	
Female	614 (62.34%)	132 (53.23%)	145 (59.43%)	175 (71.14%)	162 (65.59%)	
**BMI, kg/m2, mean (SD)**	22.37 ± 3.25	22.14 ± 3.11	22.31 ± 3.21	22.79 ± 3.36	22.23 ± 3.29	0.113
**Alcohol consumption**						0.139
Yes	152 (15.43%)	27 (10.89%)	40 (16.39%)	44 (17.89%)	41 (16.60%)	
No	833 (84.57%)	221 (89.11%)	204 (83.61%)	202 (82.11%)	206 (83.40%)	
**Smoking status**						<0.001
Yes	354 (35.94%)	61 (24.60%)	84 (34.43%)	98 (39.84%)	111 (44.94%)	
No	631 (64.06%)	187 (75.40%)	160 (65.57%)	148 (60.16%)	136 (55.06%)	
**Medical history**						
**Hypertension**	260 (26.40%)	48 (19.35%)	63 (25.82%)	68 (27.64%)	81 (32.79%)	0.008
**Diabetes**	106 (10.76%)	22 (8.87%)	26 (10.66%)	30 (12.20%)	28 (11.34%)	0.674
**CVD**	62 (6.29%)	10 (4.03%)	19 (7.79%)	17 (6.91%)	16 (6.48%)	0.355
**All-cause mortality, n (%)**						<0.001
Alive	676 (68.63%)	201 (81.05%)	180 (73.77%)	162 (65.85%)	133 (53.85%)	
Death	309 (31.37%)	47 (18.95%)	64 (26.23%)	84 (34.15%)	114 (46.15%)	
**Cancer mortality, n (%)**						<0.001
Alive	875 (88.83%)	234 (94.35%)	222 (90.98%)	213 (86.59%)	206 (83.40%)	
Death	110 (11.17%)	14 (5.65%)	22 (9.02%)	33 (13.41%)	41 (16.60%)	
**Non-cancer mortality, n (%)**						0.002
Alive	776 (78.78%)	205 (82.66%)	202 (82.79%)	195 (79.27%)	174 (70.45%)	
Death	209 (21.22%)	43 (17.34%)	42 (17.21%)	51 (20.73%)	73 (29.55%)	

NPAR, neutrophil percentage-to-albumin ratio; BMI, body mass index; CVD, cardiovascular disease.

Table S2. Cox proportional hazards regression analysis of NPAR and mortality from external validation cohort.

Exposure	Model 1<br>HR (95% CI) *P*-value	Model 2<br>HR (95% CI) *P*-value	Model 3<br>HR (95% CI) *P*-value
**All-cause mortality**			
**NPAR (continuous)**	1.05 (1.04, 1.07) <0.001	1.05 (1.04, 1.07) <0.001	1.05 (1.03, 1.07) <0.001
**NPAR quartile**			
Quartile 1	**Reference**	**Reference**	**Reference**
Quartile 2	1.36 (0.93, 1.98) 0.113	1.34 (0.92, 1.96) 0.126	1.31 (0.90, 1.91) 0.159
Quartile 3	1.80 (1.26, 2.57) 0.001	1.76 (1.23, 2.52) 0.002	1.73 (1.21, 2.49) 0.003
Quartile 4	2.79 (1.99, 3.93) <0.001	2.74 (1.95, 3.86) <0.001	2.63 (1.87, 3.71) <0.001
P for trend	<0.001	<0.001	<0.001
**Cancer mortality**			
**NPAR (continuous)**	1.06 (1.03, 1.09) <0.001	1.06 (1.03, 1.09) <0.001	1.06 (1.03, 1.09) <0.001
**NPAR quartile**			
Quartile 1	**Reference**	**Reference**	**Reference**
Quartile 2	1.57 (0.81, 3.08) 0.184	1.55 (0.79, 3.03) 0.201	1.56 (0.80, 3.06) 0.192
Quartile 3	2.37 (1.27, 4.42) 0.007	2.26 (1.20, 4.23) 0.011	2.28 (1.21, 4.28) 0.011
Quartile 4	3.33 (1.82, 6.11) <0.001	3.24 (1.76, 5.95) <0.001	3.24 (1.76, 5.98) <0.001
P for trend	<0.001	0.001	0.004
**Non-cancer mortality**			
**NPAR (continuous)**	1.04 (1.02, 1.06) 0.002	1.04 (1.02, 1.06) 0.002	1.04 (1.02, 1.06) <0.001
**NPAR quartile**			
Quartile 1	**Reference**	**Reference**	**Reference**
Quartile 2	0.97 (0.63, 1.48) 0.877	0.96 (0.63, 1.47) 0.852	0.92 (0.60, 1.42) 0.721
Quartile 3	1.20 (0.80, 1.80) 0.386	1.19 (0.79, 1.79) 0.408	1.16 (0.77, 1.75) 0.474
Quartile 4	1.97 (1.35, 2.88) <0.001	1.95 (1.33, 2.85) <0.001	1.84 (1.25, 2.70) 0.002
P for trend	<0.001	<0.001	<0.001

Model 1: No covariates were adjusted.

Model 2: Age and gender were adjusted.

Model 3: Age, gender, BMI, drinker, smoker, diabetes, hypertension, and CVD were adjusted.

Abbreviation: NPAR, neutrophil percentage-to-albumin ratio; BMI, body mass index; CVD, cardiovascular disease.

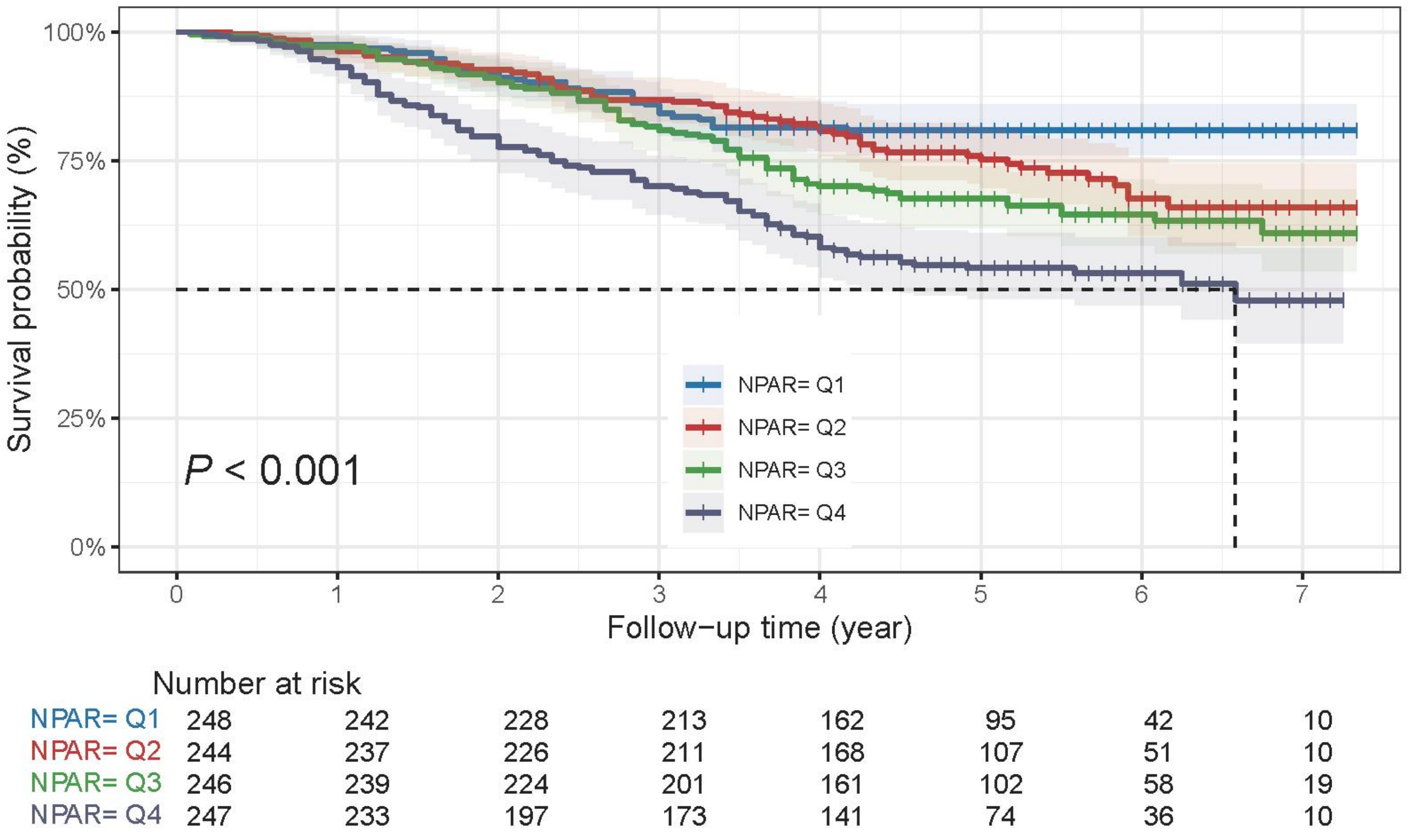

Figure S1. Kaplan-Meier curves for all-cause mortality by NPAR quartiles from external validation cohort.

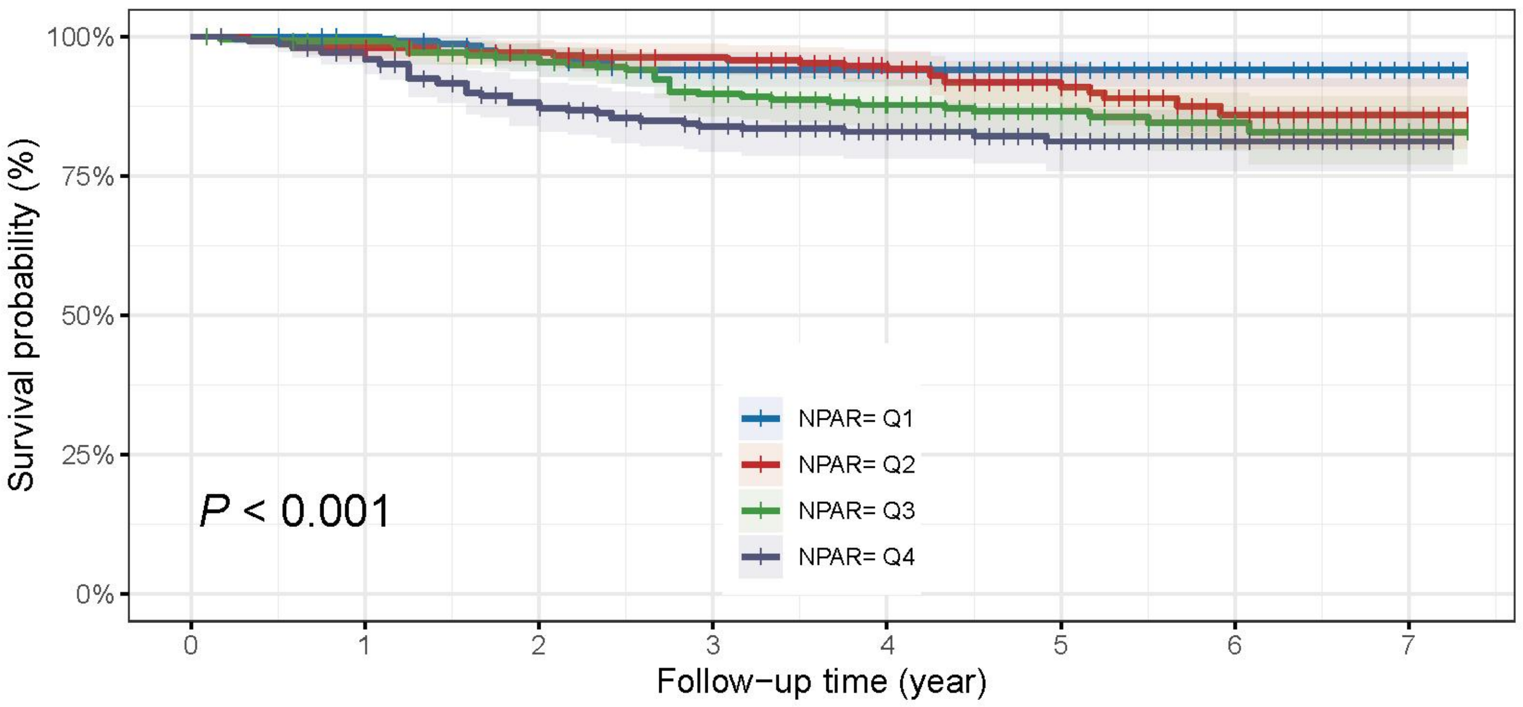

Number at risk

NPAR= Q1	248	242	228	213	162	95	42	10
NPAR= Q2	244	237	226	211	168	107	51	10
NPAR= Q3	246	239	224	201	161	102	58	19
NPAR= Q4	247	233	197	173	141	74	36	10

Figure S2. Kaplan-Meier curves for cancer-specific mortality by NPAR quartiles from external validation cohort.

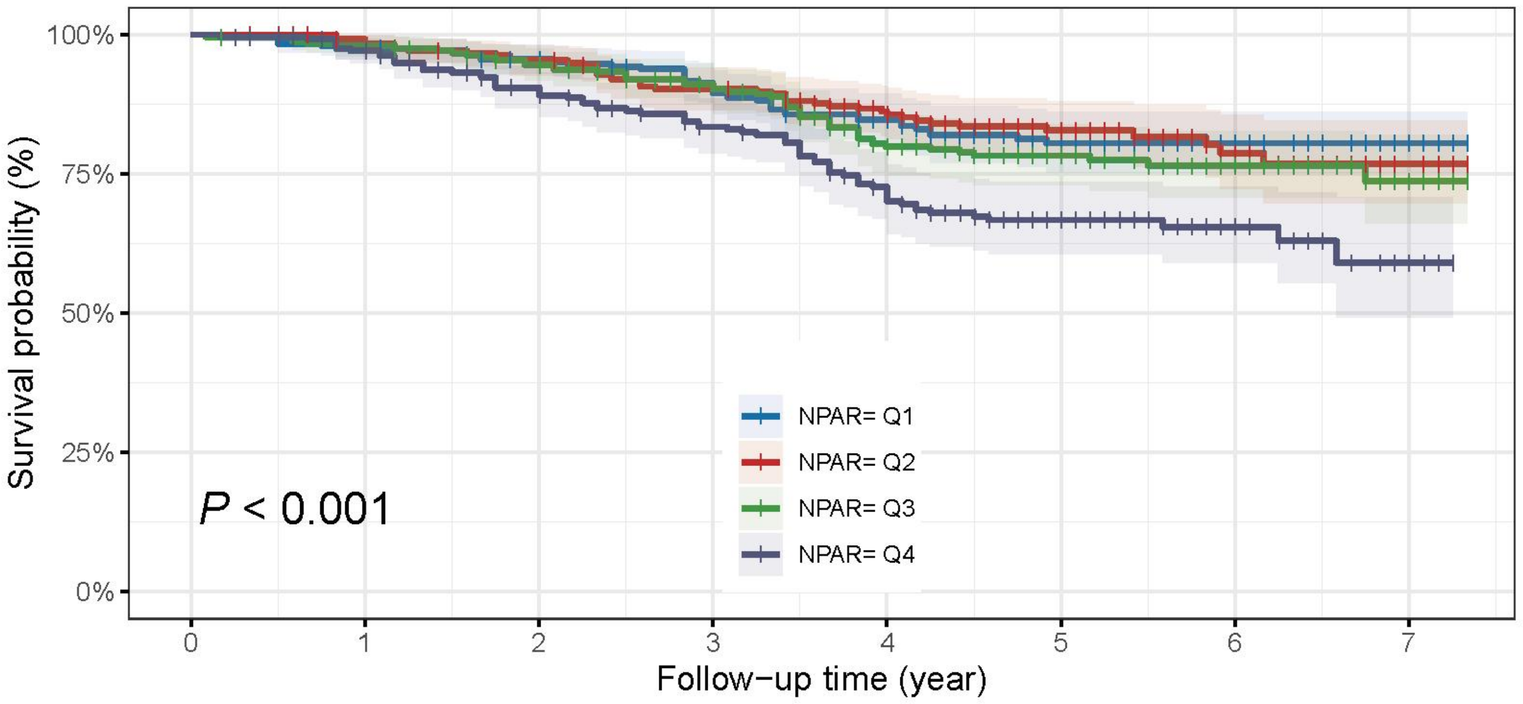

Number at risk

NPAR= Q1	248	242	228	213	162	95	42	10
NPAR= Q2	244	237	226	211	168	107	51	10
NPAR= Q3	246	239	224	201	161	102	58	19
NPAR= Q4	247	233	197	173	141	74	36	10

Figure S3. Kaplan-Meier curves for non-cancer-specific mortality by NPAR quartiles from external validation cohort.
